# Metagenomic Insights into Regional Differences in the Rhizosphere Microbial Communities of *Stellera chamaejasme* L. in Inner Mongolia

**DOI:** 10.3390/microorganisms14061167

**Published:** 2026-05-22

**Authors:** Zeyu Pan, Jian Bao, Xiangdong Liu, Gentu Ge, Muqier Zhao

**Affiliations:** 1 Key Laboratory of Forage Cultivation, Processing and Highly Efficient Utilization of Ministry of Agriculture and Rural Affairs, College of Grassland Science, Inner Mongolia Agricultural University, Hohhot 010019, China; panzeyu0520@163.com (Z.P.); 18847788933@163.com (X.L.); 2 Key Laboratory of Grassland Resources, Ministry of Education, College of Grassland Science, Inner Mongolia Agricultural University, Hohhot 010019, China; 3 College of Grassland Science, Inner Mongolia Agricultural University, Hohhot 010019, China; 4 Inner Mongolia Academy of Agricultural and Animal Husbandry Sciences, Hohhot 010031, China; baojian19940203@163.com

**Keywords:** *Stellera chamaejasme* L., rhizosphere microbiome, shotgun metagenomics, KEGG functional potential, LEfSe biomarkers, Inner Mongolian steppe

## Abstract

Rhizosphere microorganisms are important components of grassland ecosystems, but the rhizosphere microbiome of the poisonous and medicinal plant *Stellera chamaejasme* L. remains poorly characterized. In this study, shotgun metagenomic sequencing was used to compare the taxonomic composition, community structure, differentially enriched taxa, and KEGG-based functional potential of rhizosphere microbial communities associated with *S. chamaejasme* from three typical steppe regions in Inner Mongolia. Acidobacteria, Proteobacteria, and Actinobacteria were the dominant phyla, while Sphingomonas, Bradyrhizobium, and Streptomyces were among the dominant genera. Genus-level profiles and ordination analysis showed region-associated community patterns, and rarefaction curves indicated that sequencing depth was sufficient to capture most detectable taxa. LEfSe analysis identified region-associated differentially enriched taxa, including Sphingomonas-, Bradyrhizobium/Nitrospira-, and Streptomyces/Solirubrobacter-associated taxa. KEGG annotation suggested broadly similar major functional categories across regions, with some differences in the relative abundance of metabolic pathways. These results provide baseline metagenomic information on *S. chamaejasme* rhizosphere communities. Because of the limited replication and lack of soil physicochemical measurements, ecological mechanisms should be tested in future studies.

## 1. Introduction

The grassland ecosystem is one of the most important ecosystem types in the world, and its plant–microorganism interaction plays a key role in maintaining soil nutrient cycling, plant growth and ecosystem stability [1]. As the most active microenvironment of plant roots, the rhizosphere microbial community is regulated by plant species, root exudates, and local soil physical and chemical conditions [2]. Microbial communities not only participate in organic matter decomposition and nutrient cycling, but also enhance plant adaptability by producing secondary metabolites, inhibiting pathogens or regulating plant hormones [3]. Therefore, it is of great significance to systematically study the structure, taxonomic composition, and functional characteristics of rhizosphere microbial communities in grassland plants for understanding the productivity and stability of grassland ecosystems.

In recent years, the rapid development of metagenomics and high-throughput sequencing technologies has provided a powerful tool for analyzing rhizosphere microbial diversity and functional potential [4]. Metagenomic analysis can simultaneously obtain the classification information and potential functional information of microorganisms, thus revealing the coupling relationship between community structure and function. Previous studies have shown that different geographical regions, soil types, and environmental gradients can significantly affect the structure and functional potential of plant rhizosphere microorganisms [5]. For example, factors such as soil pH, water content, nutrients, and organic matter content drive the spatial distribution of dominant phyla such as Proteobacteria, Acidobacteria, and Actinobacteria, and affect nitrogen cycling, carbon metabolism, and secondary metabolism [6]. In addition, the rhizosphere microbial community showed regional optimization under different ecological conditions; that is, dominant microorganisms occupy specific niches in the local environment and maintain community stability through functional interactions [7].

*Stellera chamaejasme* L. (Thymelaeaceae), commonly known as wolf poison, is a perennial poisonous herb widely distributed in Eurasian grasslands and meadows, including northern China, Mongolia, southern Russia, and the western Himalayas. In northern China, this species often occurs in degraded grasslands and may become locally dominant under long-term grazing disturbance and grassland degradation. Although *S. chamaejasme* is toxic and generally avoided by livestock, its dried roots, known as “Rui-Xiang-Lang-Du” in traditional Chinese medicine, contain diverse bioactive compounds and have been used for medicinal purposes [8]. These ecological and chemical characteristics make *S. chamaejasme* a relevant species for studying plant–soil–microbe interactions in grassland ecosystems. Its wide distribution and strong adaptability in the northern steppe make its rhizosphere microbial community play an important role in ecological functions [8]. However, the current systematic research on the rhizosphere microorganisms of *Stellera chamaejasme* L. is still limited, especially the comparative study of community structure, core dominant bacteria and functional potential in different geographical regions [9]. Nitrogen-fixing bacteria, nitrifying bacteria, and dominant genera capable of degrading complex organic matter are considered to be key microorganisms in the rhizosphere of grassland plants to maintain the carbon and nitrogen cycle and plant growth [10]. However, differences in soil nutrients, pH, water, and vegetation in different regions may lead to significant spatial heterogeneity in the distribution and functional performance of these key microorganisms.

Previous studies have shown that the abundance and functional pathways of rhizosphere microorganisms at the phylum and genus levels in different grassland areas show regional differences. For example, *Bradyrhizobium* and *Nitrospira* are significantly enriched in areas with active nitrogen cycle, while *Streptomyces* and *Solirubrobacter* are dominant in soils with abundant organic matter or active secondary metabolism [11]. These studies have shown that the rhizosphere microbial community not only exhibits regional optimization in structure, but also its functional potential will be adjusted to environmental conditions, thereby supporting plant nutrient acquisition and stress resistance.

In this study, we used shotgun metagenomic sequencing to compare rhizosphere microbial communities associated with senescent *S. chamaejasme* plants from three typical steppe regions in Inner Mongolia. The objectives were to: (i) describe regional patterns in taxonomic composition at the phylum and genus levels; (ii) evaluate taxon richness and overall community structure; (iii) identify differentially enriched taxa among regions using LEfSe analysis; and (iv) compare KEGG-based functional potential among the three regions. Because no soil physicochemical or plant physiological measurements were included, this study focuses on descriptive regional comparisons rather than direct identification of environmental drivers or functional mechanisms.

## 2. Materials and Methods

### 2.1. Sample Collection

Rhizosphere soil samples of *Stellera chamaejasme* L. were collected in October 2023 from three typical steppe regions in Inner Mongolia, China, namely Ar Horqin Banner (AHB), Baarin Left Banner (BLB), and Baarin Right Banner (BRB). Sampling was conducted during the senescent stage of *S. chamaejasme*. The senescent stage was selected because it represents the end of the growing season in the Inner Mongolian steppe. Sampling all regions at the same phenological stage helped reduce variation associated with plant developmental differences and allowed a more consistent comparison of regional rhizosphere microbial patterns.

In each region, three visually healthy plants with no visible symptoms of damage or disease were selected as independent biological replicates. Sampled plants within each region were separated by at least 50 m to reduce local spatial autocorrelation. Each biological replicate consisted of rhizosphere soil collected from a single plant, and no soil samples, DNA extracts, or sequencing reads were pooled before analysis. Therefore, all downstream analyses were based on the individual sample-level abundance matrix.

During sampling, the soil surrounding each plant was excavated to a depth of 50–80 cm to expose the main root system of mature *S. chamaejasme* plants. This depth was chosen based on the field-observed rooting zone of the sampled plants. Loose soil on the root surface was gently shaken off, and soil tightly adhering to the roots was collected as rhizosphere soil using a disposable sterile brush. To minimize contamination, sterile gloves were worn throughout the sampling process, and all samples were collected into sterile centrifuge tubes. The collected rhizosphere soil samples were immediately frozen in liquid nitrogen in the field and then transferred to a −80 °C ultra-low-temperature freezer until further analysis.

### 2.2. DNA Extraction, Library Construction, and Sequencing

Total microbial DNA was extracted from rhizosphere soil samples using a modified CTAB-based protocol according to previously described soil DNA extraction methods. For metagenomic samples, 5–10% lysozyme was added during the lysis step to enhance microbial cell disruption. The main modification was the addition of lysozyme during lysis to improve the disruption of microbial cells in rhizosphere soil. Total microbial DNA was extracted from rhizosphere soil samples using the CTAB method. For metagenomic samples, 5–10% lysozyme was added during the lysis step to enhance microbial cell disruption. DNA concentration was measured using a Qubit 3.0 fluorometer (Invitrogen, Carlsbad, CA, USA) with the Qubit dsDNA HS Assay Kit, and DNA integrity was assessed by 1% agarose gel electrophoresis. DNA samples with a concentration of at least 1 ng/μL, a total amount of at least 0.1 μg, and a major DNA smear above 5 kb without obvious contamination were considered qualified for subsequent library construction.

For library preparation, 10 ng of genomic DNA was used as the input material. The DNA was subjected to enzymatic fragmentation and end repair, followed by adaptor and index ligation. The ligation products were purified using magnetic beads, after which the purified DNA fragments were amplified by PCR. The resulting PCR products were further size-selected using magnetic beads to obtain the target library fragments. According to the protocol provided by Biomarker Technologies, library construction was performed using the VAHTS^®^ Universal Plus DNA Library Prep Kit for Illumina, and purification was carried out using VAHTS™ DNA Clean Beads.

Library quality was evaluated using a Qsep-400 system for fragment size distribution and a Qubit 3.0 fluorometer for library quantification. Libraries meeting the following criteria were considered suitable for sequencing: concentration ≥ 1 ng/μL, fragment size center of 430–530 bp, average fragment size of 420–580 bp, and a single peak pattern without obvious nonspecific peaks. Qualified libraries were sequenced on the Illumina NovaSeq 6000 platform.

### 2.3. Sequence Quality Control, Assembly, and Gene Prediction

Raw reads were quality-filtered using Trimmomatic v0.33 with the parameters PE LEADING:3 TRAILING:3 SLIDINGWINDOW:50:20 MINLEN:120 to obtain clean reads. Clean reads were then assembled de novo using MEGAHIT v1.1.2 with default parameters, and contigs shorter than 300 bp were removed. Assembly quality was evaluated using QUAST v2.3 [12,13]. Open reading frames (ORFs) were predicted from the assembled contigs using MetaGeneMark v3.26 with default parameters [14].

To construct a non-redundant gene catalog, predicted genes from all samples were clustered using MMseqs2, with a sequence identity threshold of 95% and a coverage threshold of 90%.

### 2.4. Taxonomic Annotation and Community Composition Analysis

Taxonomic annotation was conducted based on the non-redundant gene catalog using the Nr database. The protein sequences of non-redundant genes were aligned against the Nr database using DIAMOND, with an E-value cutoff of 1 × 10^−5^, and the species information of the best-matched sequence was used for taxonomic assignment [15]. Community composition and relative abundance were summarized at different taxonomic levels. In this study, phylum-level and genus-level taxonomic profiles were used to compare rhizosphere microbial community composition among AHB, BLB, and BRB.

### 2.5. Functional Annotation

To characterize the functional potential of rhizosphere microbial communities, protein sequences predicted from the non-redundant gene catalog were annotated against the KEGG database using DIAMOND, with an E-value cutoff of 1 × 10^−5^ [16]. KEGG annotation results were used for functional classification and pathway-level comparison among the three regions.

### 2.6. Diversity and Differential Analyses

Principal component analysis (PCA) was performed to evaluate differences in microbial community structure among samples. In addition, species richness rarefaction curves were used to assess sequencing depth and genus-level richness patterns among the three groups. LEfSe analysis was conducted to identify differentially enriched taxa and potential microbial biomarkers among AHB, BLB, and BRB [17].

### 2.7. Statistical Analysis and Visualization

Taxonomic composition, PCA, rarefaction analysis, LEfSe analysis, and KEGG functional classification were conducted based on the standard metagenomic analysis workflow provided by Biomarker Technologies. Figures were generated using the corresponding analysis modules in the BMKCloud platform. Genus-level relative abundance data were used for community structure and diversity analyses. Principal component analysis (PCA) was performed to visualize differences in microbial community composition among samples. Bray–Curtis dissimilarity was calculated based on genus-level relative abundance profiles, and differences in community composition among AHB, BLB, and BRB were tested using ANOSIM with 999 permutations. Genus-level rarefaction curves were used to evaluate sequencing depth and observed genus richness. Alpha-diversity indices, including observed genera, Shannon index, and Simpson index, were calculated to describe genus-level richness and diversity. Differences among regions were tested using the Kruskal–Wallis test. A *p* value < 0.05 was considered statistically significant.

## 3. Results

### 3.1. Overall Structure and Genus-Level Richness of Rhizosphere Microbial Communities

**Figure 1:**
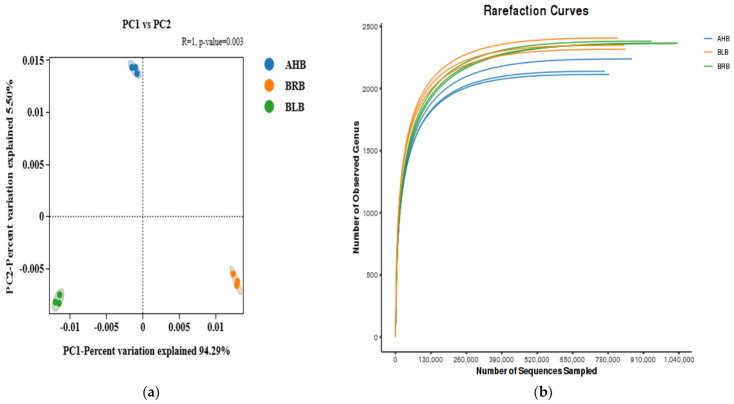
Genus-level community structure and sequencing-depth assessment of rhizosphere microbial communities of *Stellera chamaejasme* L. in AHB, BLB, and BRB. (**a**) PCA plot based on genus-level relative abundance. (**b**) Genus-level rarefaction curves showing the number of observed genera as a function of the number of sampled sequences.

Principal component analysis (PCA) was performed based on genus-level relative abundance profiles to visualize differences in rhizosphere microbial community composition among AHB, BLB, and BRB. As shown in Figure 1a, samples from the three regions showed different distribution patterns along the first two principal components. PC1 explained 94.29% of the total variation, and PC2 explained 5.50%. To statistically evaluate differences in community composition among regions, ANOSIM was performed based on Bray–Curtis dissimilarity. The results showed significant separation among the three regional groups (R = [1], *p* = [0.003]).

Genus-level rarefaction curves were used to evaluate sequencing depth and observed genus richness. As shown in Figure 1b, the rarefaction curves gradually approached a plateau as the number of sampled sequences increased, indicating that the sequencing depth was sufficient to capture most detectable genera in the samples. The asymptotic levels of the curves suggested differences in observed genus richness among regions. However, rarefaction curves were used only to assess sampling completeness and observed richness patterns, and were not used as direct evidence of community stability, dominance, or functional complexity.

Alpha-diversity indices were further used to evaluate richness and diversity differences among regions. The results showed that observed taxon richness differed among the three groups (Kruskal–Wallis test, H = 6.006, *p* = 0.0496), and Shannon diversity also showed significant group-level differences (H = 7.200, *p* = 0.0273). In contrast, ACE, Chao1, and Simpson indices did not differ significantly among groups at the *p* < 0.05 level (ACE: H = 5.956, *p* = 0.0509; Chao1: H = 5.956, *p* = 0.0509; Simpson: H = 5.422, *p* = 0.0665). Good’s coverage values were close to 1.0 in all samples, indicating sufficient sequencing coverage. Therefore, the rarefaction analysis supports the adequacy of sequencing depth and provides an overview of observed genus richness, but conclusions regarding significant richness differences should be based on alpha-diversity statistics rather than rarefaction curves alone.

### 3.2. Rhizosphere Microbial Community Composition and Taxonomic Differences of Stellera chamaejasme L.

**Figure 2:**
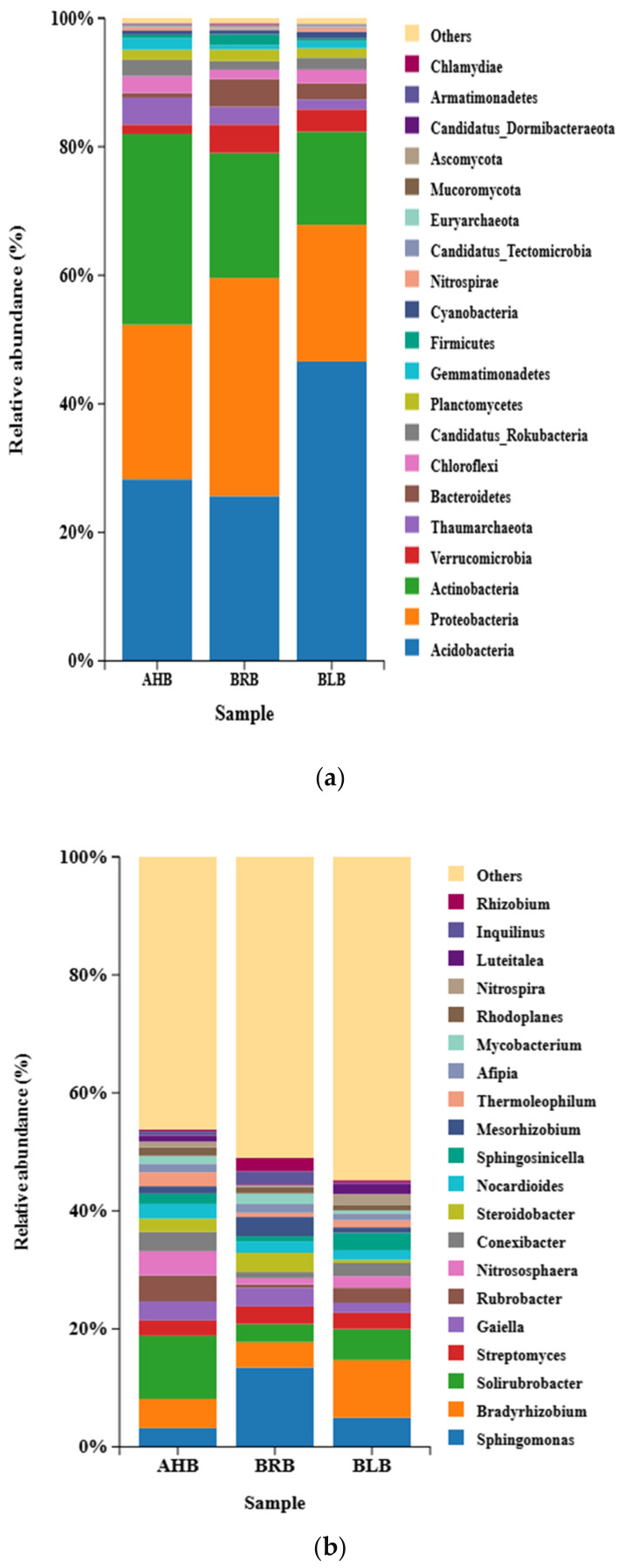
Sample-level taxonomic composition and relative abundance of rhizosphere microbial communities associated with *Stellera chamaejasme* L. in AHB, BLB, and BRB. (**a**) Stacked bar plot showing the relative abundance of the top 20 bacterial phyla across the nine biological replicates. (**b**) Stacked bar plot showing the relative abundance of the top 20 bacterial genera across the nine biological replicates. Taxa outside the top 20 were grouped as “Others”.

At the phylum level, Acidobacteria, Proteobacteria, and Actinobacteria were the dominant bacterial phyla across the three regions (Figure 2a). The sample-level relative abundance profiles showed region-associated differences in dominant phyla. Based on the verified abundance values, Acidobacteria was relatively enriched in BLB, Proteobacteria was relatively enriched in BRB, and Actinobacteria was relatively enriched in AHB. Other phyla, including Thaumarchaeota, Bacteroidetes, and Verrucomicrobia, contributed to the lower-abundance components of the rhizosphere microbial communities. These patterns describe differences in taxonomic composition among regions, but they should not be interpreted as direct evidence of functional differences without further validation.

At the genus level, Sphingomonas, Bradyrhizobium, and Streptomyces were among the dominant genera detected in the rhizosphere microbial communities (Figure 2b). Sphingomonas was relatively enriched in [insert group], Bradyrhizobium was relatively enriched in [insert group], and Streptomyces was relatively enriched in [insert group]. These genus-level profiles indicate region-associated differences in taxonomic composition. However, functional implications of these taxa should be interpreted cautiously because taxonomic identity alone does not directly demonstrate functional activity.

### 3.3. Identification of Biomarkers for Distinguishing Different Groups of Stellera chamaejasme L. Rhizosphere Microorganisms

**Figure 3:**
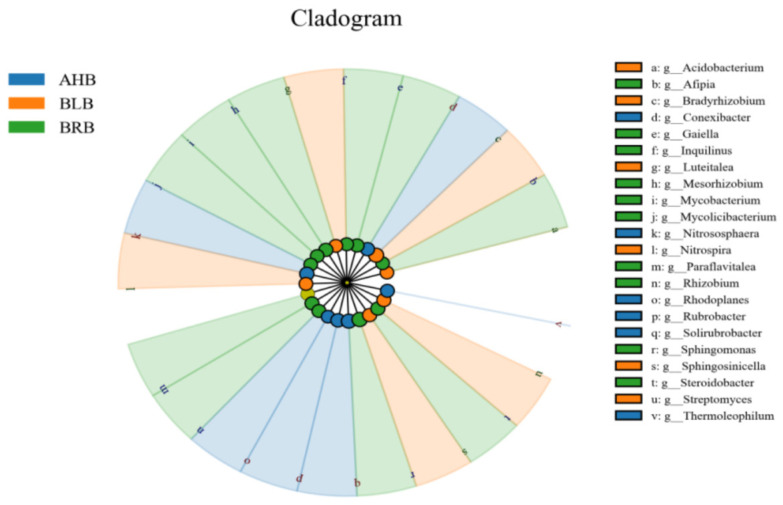
Cladogram analysis of rhizosphere microbial genera in different groups (AHB/BLB/BRB).

The results showed that 5, 5, and 10 biomarkers were identified in AHB, BLB and BRB groups, respectively (Figure 3). At the genus level, the specific enrichment of the AHB group mainly includes: g_*Sphingomonas* and g_*Afipia*, g_*Mesorhizobium*, etc. This is completely consistent with the results observed in the previous relative abundance map. The characteristic genera specifically enriched in the BRB group were very prominent, including g_*Bradyrhizobium*, g_*Nitrospira*, g_*Acidobacterium*, etc. These groups are also highly consistent with the characteristics of BRB in the relative abundance map and PCA analysis. The BLB group shows unique enrichment genera, such as g_*Gaiella*, g_*Solirubrobacter*, g_*Streptomyces* and g_*Thermoleophilum*. This indicates that BLB is not a simple mixture of AHB and BRB, but has its own specific microbial groups.

**Figure 4:**
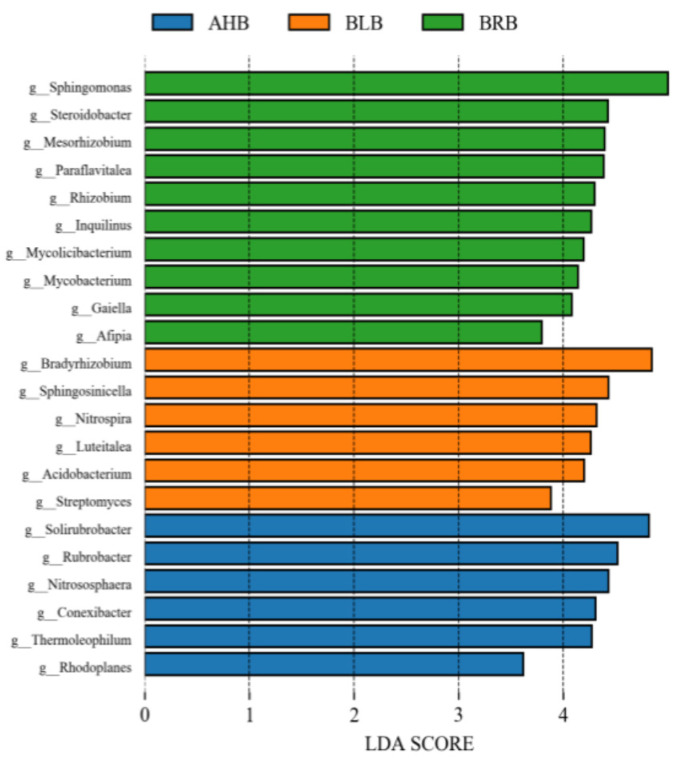
LDA analysis of biomarkers of different groups (AHB/BLB/BRB) of *Stellera chamaejasme* L. rhizosphere microorganisms.

The LDA value distribution of LEfSe analysis further quantified the discriminant power of specific biomarkers in each group (Figure 4). The results showed that the most discriminative biomarker in the AHB group was g_*Sphingomonas*, with the highest LDA value (close to 4), followed by g_*Mesorhizobium* and so on. In the BRB group, the LDA values of g_*Bradyrhizobium* and g_*Nitrospira* were very prominent (both greater than 3.5), which were the key markers to distinguish BRB. *Acidobacterium* g_(*Acidobacterium*) also has a high discriminant power. In the BLB group, g_*Streptomyces* and g_*Solirubrobacter* were the most discriminative markers (LDA value > 3). These groups with high LDA values are the most critical microbial characteristics to distinguish the three groups of samples.

### 3.4. Comparison of Average Relative Abundance of Key Rhizosphere Microbial Communities in Stellera chamaejasme L.

**Figure 5:**
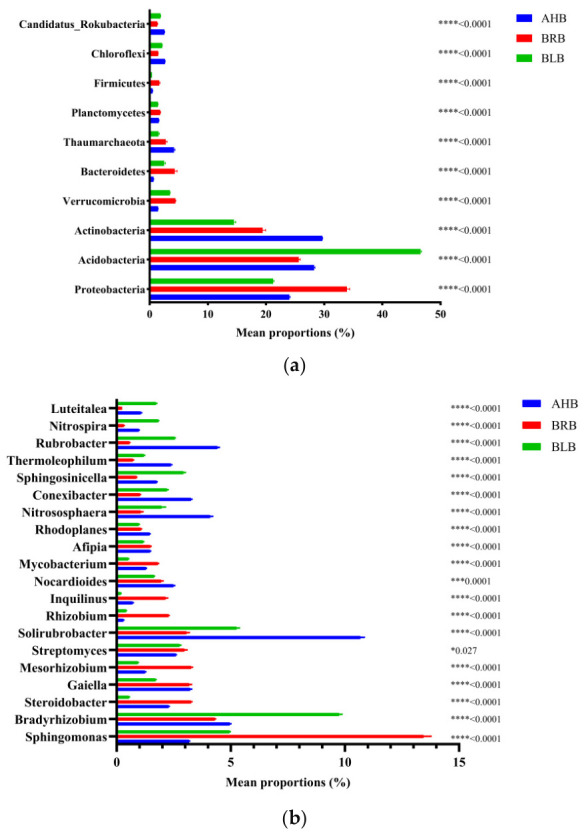
The differences in the mean abundance of the main bacterial phyla in the three groups of samples (AHB, BRB, BLB) can intuitively compare the overall distribution pattern of different microbial groups between groups. (**a**) The average relative abundance map of the level of gate. (**b**) The average relative abundance map of genus level.

Figure 5a shows that the three groups of samples have significantly different and specifically enriched core flora at the phylum level. Proteobacteria and Acidobacteria were the two most abundant phyla in all groups, but their dominance was opposite between groups. In the AHB group, the average abundance of Proteobacteria was the highest, while the abundance of Acidobacteria was relatively low. In the BRB group, the situation was completely opposite. The average abundance of Acidobacteria was the highest, while the abundance of Proteobacteria was significantly lower than that of AHB. In the BLB group, the abundance of these two phyla was between AHB and BRB, but closer to the pattern of BRB (i.e., the abundance of Acidobacteria was slightly higher or comparable to that of Proteobacteria). Actinobacteria showed the highest average abundance in the BLB group, which was consistent with its identification as an iconic group of BLB in LEfSe analysis. The average abundance of Bacteroidetes and Verrucomicrobia in the AHB group was higher than that in the other two groups. The average abundance of Thaumarchaeota in the BRB group was significantly higher.

Figure 5b shows that the three groups of samples have significantly different and specifically enriched core flora at the genus level. The average relative abundance of *Sphingomonas* was the highest, which was the core dominant bacteria of AHB. *Mesorhizobium*, *Afipia*, *Rhizobium*, and *Bradyrhizobium* (higher abundance in AHB, but higher in BRB) maintained high abundance in AHB. The average relative abundance of *Bradyrhizobium* in BRB was the highest, which was the core marker bacteria of BRB. The abundance of *Nitrospira* in BRB was significantly higher than that in the other two groups. *Acidobacterium* and *Rhodoplanes* also have high abundance in BRB. The average abundance of *Streptomyces* and *Solirubrobacter* in BLB was significantly higher than that of the other two groups, which were the characteristic genera of this group. *Gaiella* and *Thermoleophilum* were also relatively enriched in BLB. The abundance of *Bradyrhizobium* in both AHB and BRB was high, but the degree of dominance was different, suggesting that it may be a key functional bacterium that is widely present but regulated by the environment. *Nitrospira* and *Nitrososphaera*, as nitrifying functional bacteria, are more prominent in BRB, suggesting that the nitrogen cycle in this group is active.

### 3.5. Prediction of Microbial Function of Stellera chamaejasme L. Root System

**Figure 6:**
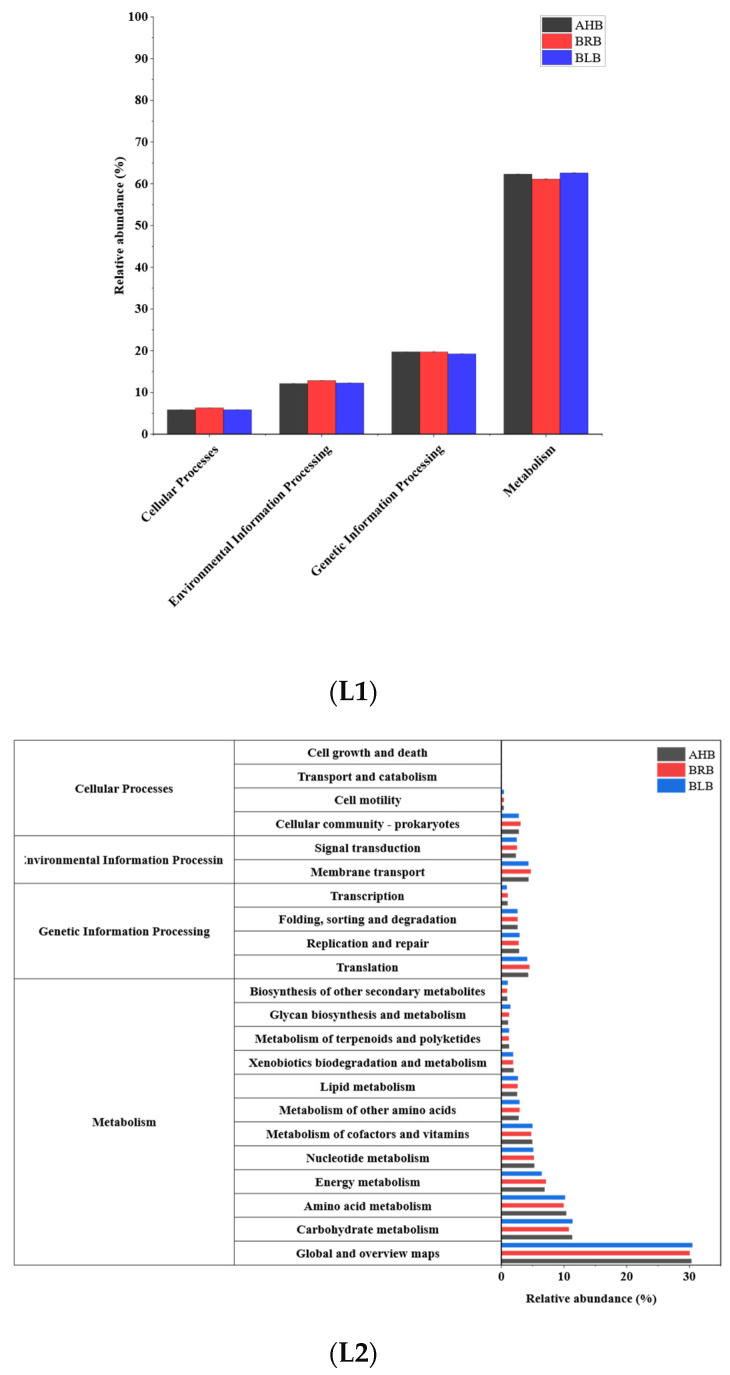

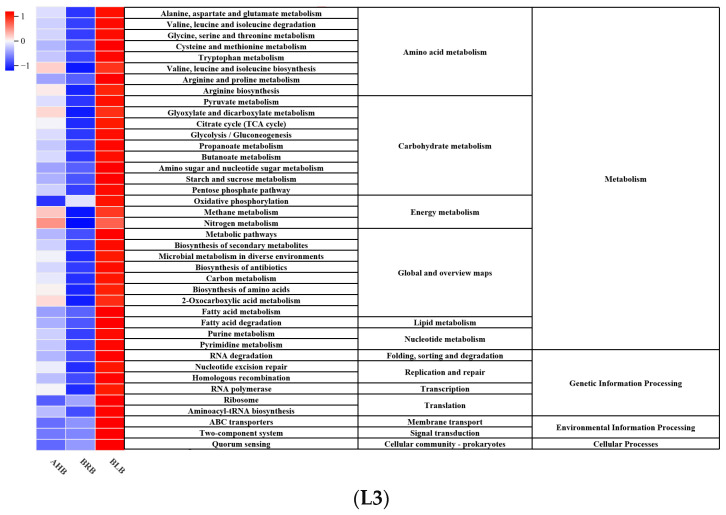
Analysis of KEGG functional annotation characteristics of rhizosphere microbial community of *Stellera chamaejasme* L. in different regions (**L1**) The relative abundance of KEGG first-order functional classification of rhizosphere microbial communities in different regions of *Stellera chamaejasme* L. (**L2**) The relative abundance of KEGG secondary functional classification of rhizosphere microbial communities in different regions of *Stellera chamaejasme* L. (**L3**) KEGG functional pathway abundance heat map of rhizosphere microbial community of *Stellera chamaejasme* L. in different regions.

As shown in Figure 6 KEGG annotation was used to compare the inferred functional potential of rhizosphere microbial communities among the three regions. At the KEGG L1 level, metabolism represented the largest functional category in all groups, followed by genetic information processing and environmental information processing. The overall distribution of KEGG L1 categories was broadly similar among AHB, BLB, and BRB, suggesting that the major functional potential categories were conserved across regions.

At the KEGG L2 level, global and overview maps, carbohydrate metabolism, amino acid metabolism, energy metabolism, nucleotide metabolism, and metabolism of cofactors and vitamins were the major categories. Differences in relative abundance were observed among some categories, but these patterns should be interpreted as differences in annotated functional potential rather than evidence of actual pathway activity.

At the KEGG L3 level, selected metabolic pathways showed region-associated differences in relative abundance. However, because metagenomic KEGG annotation does not measure gene expression, enzyme activity, or biogeochemical process rates, these results cannot directly demonstrate active nitrogen cycling, plant defense, or environmental response. They provide hypotheses for future validation using metatranscriptomic, metabolomic, or targeted functional assays.

## 4. Discussion

### 4.1. Differences in the Overall Structure and Diversity of Microbial Communities in the Rhizosphere of Stellera chamaejasme L.

This study showed region-associated differences in the rhizosphere microbial community composition of *S. chamaejasme* across AHB, BLB, and BRB. PCA provided an exploratory visualization of sample distribution patterns, while alpha-diversity indices were used to statistically evaluate richness and diversity. Rarefaction curves and Good’s coverage values indicated that sequencing depth was sufficient to capture most detectable taxa in the samples.This is consistent with the spatial distribution pattern of three samples in PCA, suggesting that there is a consistency between microbial community diversity and overall composition characteristics [18].

These differences may be influenced by a variety of environmental factors. Geographical location and soil properties are widely considered to be important factors driving rhizosphere microbial community structur [19].The alpha-diversity results showed group-level differences in observed taxon richness and Shannon diversity, whereas ACE, Chao1, and Simpson indices did not differ significantly among groups at the *p* < 0.05 level. These results suggest that some aspects of richness and diversity varied among groups, but they do not support broad claims regarding community complexity, stability, or dominance. Because soil physicochemical properties and environmental covariates were not measured, the drivers of these regional patterns cannot be directly determined from the present datasetIn addition, the differences in community structure may also be related to the differences in plant secretions and local ecological conditions, which is consistent with previous studies on the selective shaping of rhizosphere microbial communities by plants in different ecosystems [20].

### 4.2. Differences in Microbial Community Composition and Taxonomy of Stellera chamaejasme Rhizosphere L.

In the rhizosphere microbial community of *Stellera chamaejasme* L., the phylum-level classification showed that Acidobacteria, Proteobacteria, and Actinobacteria were dominant, and their relative abundance was significantly different among different regions (Figure 2a) [21, 22]. This distribution pattern is consistent with previous reports of dominant bacterial phyla in the plant rhizosphere system, indicating that Proteobacteria, Acidobacteria, and Actinobacteria are usually the core groups in the rhizosphere ecosystem [23,24]. In this study, Proteobacteria had the highest abundance in AHB, while Acidobacteria was dominant in BRB, which may reflect the effects of environmental gradients such as soil nutrient status, pH and root exudates on the niche selection of different bacterial phyla [25]. The relative enrichment of Actinobacteria in BLB suggests that there may be ecological conditions conducive to its growth in this region, such as higher organic carbon or trace element supply, which is consistent with the reports that Actinobacteria have high adaptability in soils rich in complex organic matter [26].

The distribution of secondary groups such as Thaumarchaeota, Bacteroidetes, and Verrucomicrobia in different samples showed obvious specificity, which may reflect the sensitive response of these groups to specific microenvironment factors. For example, for Thaumarchaeota, as a typical ammonia-oxidizing archaea, its high abundance in BRB may be related to the enhancement of nitrogen cycle in the region. Similar reports that ammonia-oxidizing microorganisms affect nitrogen dynamics in the rhizosphere have been verified in various systems [27].

At the genus level, *Sphingomonas*, *Bradyrhizobium*, and *Streptomyces* were the dominant genera of rhizosphere microbial communities in the three regions (Figure 2b). *Sphingomonas* often occurs in rhizosphere microbial communities of different plants, and is considered to be able to participate in the degradation of complex organic matter, promote plant growth, and regulate community structure by interacting with plants [28]; *streptomyces* is distributed in all samples, which is known to be involved in the production of antimicrobial compounds and pathogen inhibition, and may play a role in maintaining the health of the rhizosphere of *Stellera chamaejasme* L. [29]. The BLB sample showed an "intermediate" community structure at the genus level, and its abundance distribution was between AHB and BRB, which, together with PCA and diversity analysis, pointed to the sample as a transitional community. Based on the above results, this study revealed significant differences in the composition of microbial communities in the rhizosphere of *Stellera chamaejasme* L. in different regions, which was not only reflected in the relative abundance of dominant phyla and genera, but also in the spatial distribution of low-abundance functional groups. These differences may be co-shaped by local soil physical and chemical properties, rhizosphere matrix, and vegetation-driven selective factors, which provides a basis for subsequent functional prediction and environmental driving mechanism analysis.

### 4.3. Biomarker Identification of Rhizosphere Microorganisms in Different Groups of Stellera chamaejasme L.

In this study, specific biomarkers of *Stellera chamaejasme* L. rhizosphere microbial communities in different regions were identified by LEfSe analysis. The results showed that 5, 5, and 10 specific genera were enriched in AHB, BLB, and BRB samples, respectively (Figure 3 and Figure 4). These markers were highly consistent with the previous relative abundance and PCA analysis results, which verified the reliability of distinguishing each group of communities.

In AHB, *Sphingomonas*, *Afipia*, and *Mesorhizobium* were identified as the main biomarkers, among which *Sphingomonas* had the highest LDA value, suggesting that it had important ecological functions in the rhizosphere of the region. *Sphingomonas* bacteria have been reported to be able to participate in the degradation of complex organic matter, promote plant growth and tolerate various environmental stresses. Its enrichment in AHB may enhance the functional diversity of rhizosphere and improve community stability [30]. *Mesorhizobium* is a typical rhizosphere nitrogen-fixing symbiotic bacterium, and its specific enrichment in AHB samples may reflect the local demand for nitrogen cycling in this region. Nitrogen-fixing microorganisms such as *Mesorhizobium* can establish nitrogen-fixing interactions with plant roots, convert atmospheric nitrogen into available nitrogen, and improve nitrogen supply [31].

In BRB samples, *Bradyrhizobium*, *Nitrospira*, and *Acidobacterium* were the key markers of enrichment, and their LDA values were all more than 3.5, showing strong discrimination. The function of *Bradyrhizobium* in nitrogen fixation and plant growth promotion is widely recognized, and for *Nitrospira*, as a key nitrification microorganism, its enrichment in BRB may indicate an active nitrogen cycle in the region [32]. The high abundance of *Acidobacterium* may also be related to the adaptability of soil acidic conditions and nutrient-limited environments in the region [33].

BLB samples showed unique specific markers, such as *Streptomyces*, *Solirubrobacter*, and *Thermoleophilum*, with LDA values greater than 3, showing significant discriminant ability. *Streptomyces* and *Solirubrobacter* are known to produce a variety of antimicrobial compounds and participate in the decomposition of soil organic matter, which may give the BLB sample community higher functional potential and ecological adaptability [34]. *Thermoleophilum* is a thermophilic and oleophilic microorganism, and its enrichment suggests that there may be specific microenvironment selection pressure in the rhizosphere of BLB, which makes its microbial community present unique functional attributes. Overall, LEfSe analysis revealed key biomarkers for three groups of *S. chamaejasme* rhizosphere microbial communities. These high LDA-value genera can not only effectively distinguish samples from different regions, but also reflect the regulatory effect of regional environmental conditions on the functional structure of the community. This indicates that the geographical differentiation of rhizosphere microbial communities is not only reflected in the overall composition and diversity, but also in the enrichment pattern of key functional microorganisms, which provides a basis for further functional prediction and ecological mechanism analysis.

### 4.4. KEGG-Based Functional Potential and Limitations of Functional Inference

Based on the metagenomic data of rhizosphere microorganisms from different sources of *Stellera chamaejasme* L., this study revealed significant differences in rhizosphere microbial community structure at the taxonomic level among the three sampling sites (AHB, BLB, BRB), indicating that geographical environment and soil properties may shape specific microbial composition and functional potential.

At the phylum level, Proteobacteria and Acidobacteria, as the dominant bacteria in most soil ecosystems, occupy a high abundance in the three groups of this study, indicating that they may be the core members of the *Stellera chamaejasme* L. rhizosphere flora [35]. However, the two showed significant differences in abundance patterns in samples from different regions. Proteobacteria dominated in the AHB group, while Acidobacteria dominated in the BRB group, which may reflect the different responses of different soil nutrient status and pH values to these two phyla [36]. Previous studies have pointed out that Proteobacteria are generally enriched in soils rich in available carbon sources, while Acidobacteria are more suitable for low-nutrient and acidic environments [37]. This ecological explanation may partially explain this result. Actinobacteria was significantly enriched in the BLB group, which was consistent with the results of LEfSe-assisted analysis. This group is usually related to soil organic matter decomposition and stress resistance [38], which may reflect that the BLB soil has unique organic material characteristics or long-term drought stress background, thus selectively enriching such functional dominant bacteria. In addition, the higher abundance of Bacteroidetes and Verrucomicrobia in AHB samples may be related to the release of easily degradable substrates in the rhizosphere, as these phyla are often associated with carbon cycle and complex carbon source degradation [39]. The high abundance of Thaumarchaeota in BRB suggests that the potential nitrogen cycle functional activity is enhanced, because this archaea group is closely related to the ammonia oxidation process [40].

At the genus level, *Sphingomonas* was significantly enriched in the rhizosphere of AHB, which was considered to play an important role in the rhizosphere ecosystem due to its ability to degrade complex compounds such as polycyclic aromatic hydrocarbons [41]. At the same time, the high abundance of *Mesorhizobium*, *Afipia*, *Rhizobium*, and *Bradyrhizobium* in AHB and BRB may be related to the plant–microbe symbiotic nitrogen fixation function, indicating that these rhizobia may play a key role in the rhizosphere symbiotic network of *Stellera chamaejasme* L. [42]. In particular, *Bradyrhizobium* is present in all three places, but it is more prominent in BRB, indicating its sensitivity to specific environmental factors and the importance of ecological functions. The high abundance of *Nitrospira* and *Nitrososphaera* in BRB samples further suggested that the rhizosphere microbial community in this area had high nitrification potential, which may strengthen the soil nitrogen transformation process, consistent with the environmental driving theory of nitrogen cycling functional community [43]. In BLB, the enrichment of *Streptomyces* and *Solirubrobacter* re-emphasized the uniqueness of this group. As an important producer of soil-derived antibiotics, *Streptomyces* has potential benefits in inhibiting pathogenic microorganisms and promoting plant health [44], while *Solirubrobacter* is often associated with complex organic matter decomposition. These differentiated community structures not only reflect the selective pressure of geographical environment and soil physical and chemical properties, but also may be related to the differences in the composition of root exudates of *Stellera chamaejasme* L., thus affecting the functional distribution of rhizosphere microorganisms. The remodeling of rhizosphere symbiotic network may further affect plant nutrient absorption, stress resistance, and ecological adaptability. In the future, it is necessary to combine soil physical and chemical indicators and functional metabolism analysis to further clarify the specific effects of these microbial community differences on the ecological function of *Stellera chamaejasme* L.

### 4.5. Limitations and Future Directions

The L1-level functional prediction showed that the overall structure of the microbial community in the rhizosphere of *Stellera chamaejasme* L. in the three places was highly conserved in the four functional categories (metabolism, genetic information processing, environmental information processing, and cellular processes). The metabolic function was absolutely dominant (>60%), genetic information processing accounted for about 20%, environmental information processing accounted for 12–13%, and cellular processes accounted for the smallest proportion (6–7%). This functional structure pattern is consistent with previous studies on soil microorganisms in arid and semi-arid grasslands, indicating that the rhizosphere microbial community is mainly centered on maintaining basal metabolic activities [45]. In the three samples, the relative abundance of BRB in environmental information processing and cellular processes was slightly higher, suggesting that the microbial community in the region may have stronger environmental perception and response capabilities, which may be related to local soil nutrient limitations or microclimate differences. The metabolic function of AHB samples is relatively the highest, and the abundance of carbon and nitrogen cycle-related pathways in particular is higher, which may reflect the core role of rhizosphere microorganisms in maintaining plant nutrient absorption and energy conversion in the region [46].

In the L2 function prediction, the metabolic function is still the core, and the core secondary pathways mainly include global and overview maps, carbohydrate metabolism, and amino acid metabolism, showing the consistency and stability of the rhizosphere microorganisms of *Stellera chamaejasme* L. in carbon and nitrogen metabolism. This is consistent with the basic nutrient cycling function of rhizosphere microorganisms in plant rhizosphere ecosystems [18]. In terms of inter-group differences, the energy metabolism of BRB samples was slightly higher, AHB was slightly higher in the pathway related to xenobiotic degradation, and the relative abundance of BLB in secondary metabolites and terpenoids and polyketides metabolism was slightly higher, suggesting that the soil environment and plant–microorganism interactions in different regions may drive the fine-tuning of microbial functions [47].

Further L3-level analysis showed that there were some differences in specific metabolic pathways among the three samples. For example, BRB is most prominent in alanine, aspartic acid, glutamic acid and arginine and proline metabolism, TCA cycle, oxidative phosphorylation, and nitrogen metabolism, which echoes its high abundance of nitrifying functional bacteria in the rhizosphere community (such as *Nitrospira* and *Nitrososphaera*), indicating that the nitrogen cycle may be more active in the BRB rhizosphere [48]. Glycine, serine, threonine, and tryptophan metabolism, as well as fatty acid metabolism, were relatively enriched in AHB samples, suggesting that the microbial community may have advantages in carbon and nitrogen interaction metabolism and energy storage. BLB samples had the highest relative abundance in arginine biosynthesis, starch and sucrose metabolism, secondary metabolites, and antibiotic synthesis pathways, which was consistent with the enrichment of its core genera (such as *Streptomyces* and *Solirubrobacter*), suggesting that the microbial community in this area may play a greater role in plant defense, disease resistance and adaptation to environmental stress [49].

In general, the rhizosphere microorganisms of *Stellera chamaejasme* L. maintained stable basic metabolic functions in the three places, but the soil environment and plant–microbe interactions in different regions fine-tuned the metabolic potential of microorganisms. BRB rhizosphere microorganisms tend to nitrogen cycle and energy metabolism, AHB focuses more on carbon-nitrogen interaction and basal metabolism, and BLB has advantages in secondary metabolism and the production of anti-bioactive substances, which indicates that the distribution of microbial functions may optimize the plant–microbial ecological interaction in different environmental backgrounds. The sampling intensity should be considered when interpreting the results. Although each region included three independent plant-level biological replicates and each replicate was sequenced separately, the sample size was lower than that of many plot-based rhizosphere microbial studies. Therefore, the present study should be regarded as an exploratory metagenomic comparison rather than a complete assessment of regional microbial heterogeneity. Future studies should include more plants, multiple plots within each region, soil physicochemical measurements, and vegetation data to better identify the environmental drivers of *S. chamaejasme* rhizosphere microbial communities.

## 5. Conclusions

Metagenomic profiling revealed region-associated differences in the rhizosphere microbial communities of *Stellera chamaejasme* L. from three typical steppe regions in Inner Mongolia. Acidobacteria, Proteobacteria, and Actinobacteria were the dominant phyla, while Sphingomonas, Bradyrhizobium, and Streptomyces were among the dominant genera. LEfSe analysis identified differentially enriched taxa among regions, providing candidate microbial markers for distinguishing the three rhizosphere community profiles.

KEGG annotation showed that the major functional categories were broadly similar across regions, with metabolism-related categories representing the largest proportion of annotated functions. Some KEGG pathways differed in relative abundance among regions, suggesting differences in inferred functional potential. However, these results should be interpreted cautiously because of the limited sample size, lack of complete environmental metadata, and absence of functional validation. Future studies with larger sampling designs, soil physicochemical measurements, and transcriptomic or metabolomic validation are needed to clarify the ecological mechanisms underlying *S. chamaejasme* rhizosphere microbial patterns.

## Data Availability

The raw metagenomic sequencing data generated in this study have been deposited in the NCBI Sequence Read Archive (SRA) under BioProject accession number PRJNA1441840 and are publicly accessible at https://www.ncbi.nlm.nih.gov/sra/PRJNA1441840.

## Abbreviations

The following abbreviations are used in this manuscript:

BLB	Baarin Left Banner
BRB	Baarin Right Banner
AHB	Ar Horqin Banner
